# Supplementary material for: Summarizing performance for genome scale measurement of miRNA: reference samples and metrics
Source: BMC Genomics. 2018 Mar 6;19:180. doi: 10.1186/s12864-018-4496-1 (PMC5838960; doi:10.1186/s12864-018-4496-1)

Additional File 1

Measurement Process A
Round 3

Class	Detected	Median	IQR	Component	Mix1	Mix2	Bias
1–to–1*	56	−0.0326	0.237	Liver	0.12	0.118	0.185
Brain	103	−0.0348	0.248	Brain	0.257	0.525	0.026
Placenta	104	0.0406	0.150	Placenta	0.623	0.357	0.163
NS	928	−0.0159	0.349				
All	1191	−0.0133	0.303	All			0.375

LowerLimit	Maximum	Range	AUC (All)	AUC (Range)
5.77	20.02	14.25	0.959	0.996

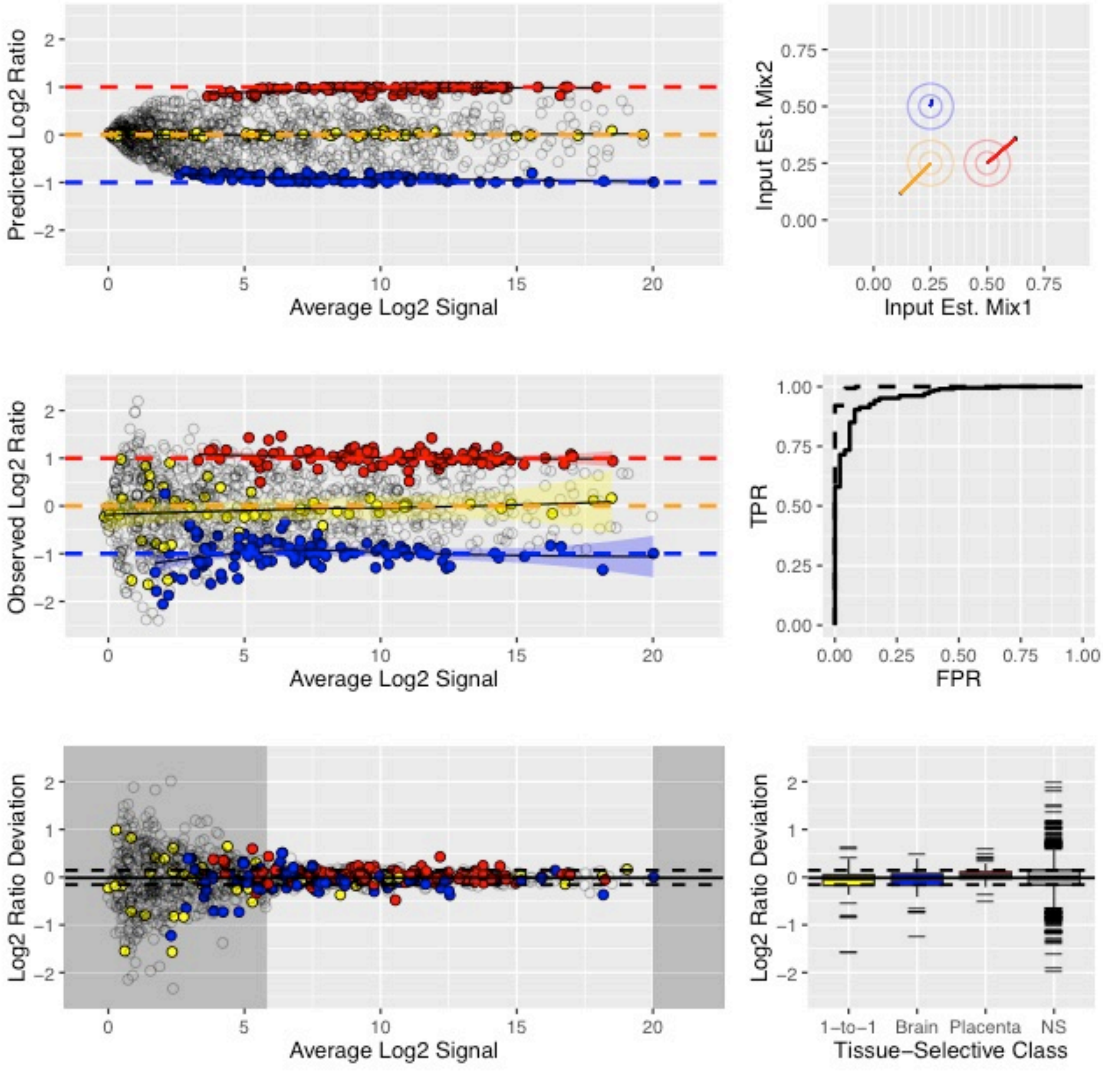

Measurement Process A
Round 4

Class	Detected	Median	IQR	Component	Mix1	Mix2	Bias
1-to-1*	72	−0.0088	0.342	Liver	0.118	0.116	0.189
Brain	101	−0.0512	0.205	Brain	0.251	0.523	0.023
Placenta	105	0.0638	0.199	Placenta	0.632	0.361	0.172
NS	935	−0.0014	0.351				
All	1213	0.0071	0.309	All			0.384

LowerLimit	Maximum	Range	AUC (All)	AUC (Range)
5.7	20.23	14.54	0.977	0.99

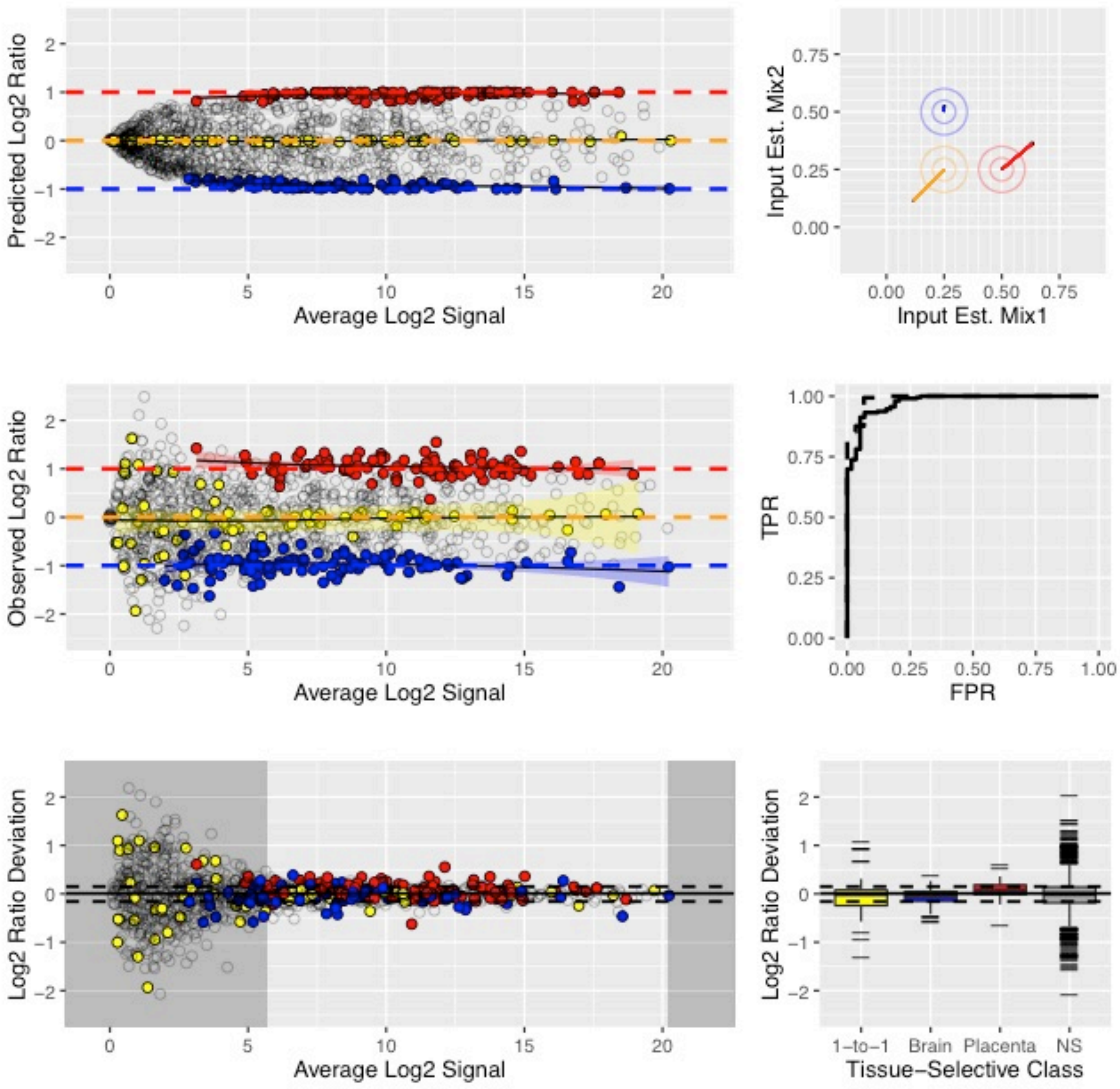

Measurement Process A
Round 5

Class	Detected	Median	IQR	Component	Mix1	Mix2	Bias
1−to−1*	59	0.0071	0.288	Liver	0.136	0.117	0.175
Brain	107	−0.0464	0.238	Brain	0.256	0.53	0.031
Placenta	105	−0.0634	0.156	Placenta	0.608	0.352	0.149
NS	859	−0.0594	0.441				
All	1130	−0.0533	0.373	All			0.355

LowerLimit	Maximum	Range	AUC (All)	AUC (Range)
6.34	20.25	13.91	0.909	0.963

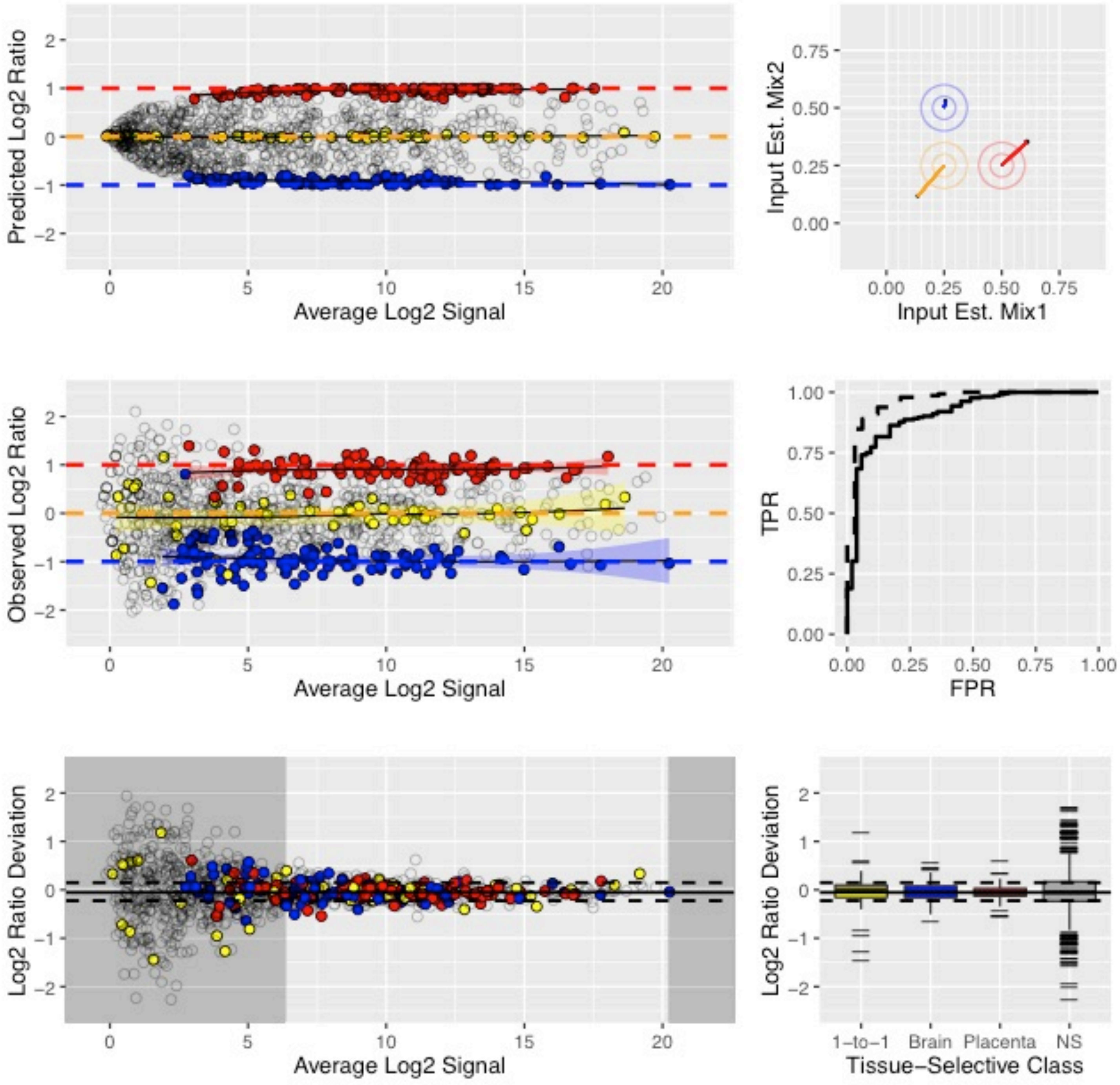

Measurement Process B
Round 3

Class	Detected	Median	IQR	Component	Mix1	Mix2	Bias
1−to−1*	56	−0.1031	0.404	Liver	0.094	0.128	0.198
Brain	134	−0.1271	0.279	Brain	0.191	0.493	0.059
Placenta	47	−0.0911	0.194	Placenta	0.715	0.38	0.251
NS	868	−0.0742	0.425				
All	1105	−0.0850	0.387	All			0.508

LowerLimit	Maximum	Range	AUC (All)	AUC (Range)
7.34	20.15	12.81	0.905	0.953

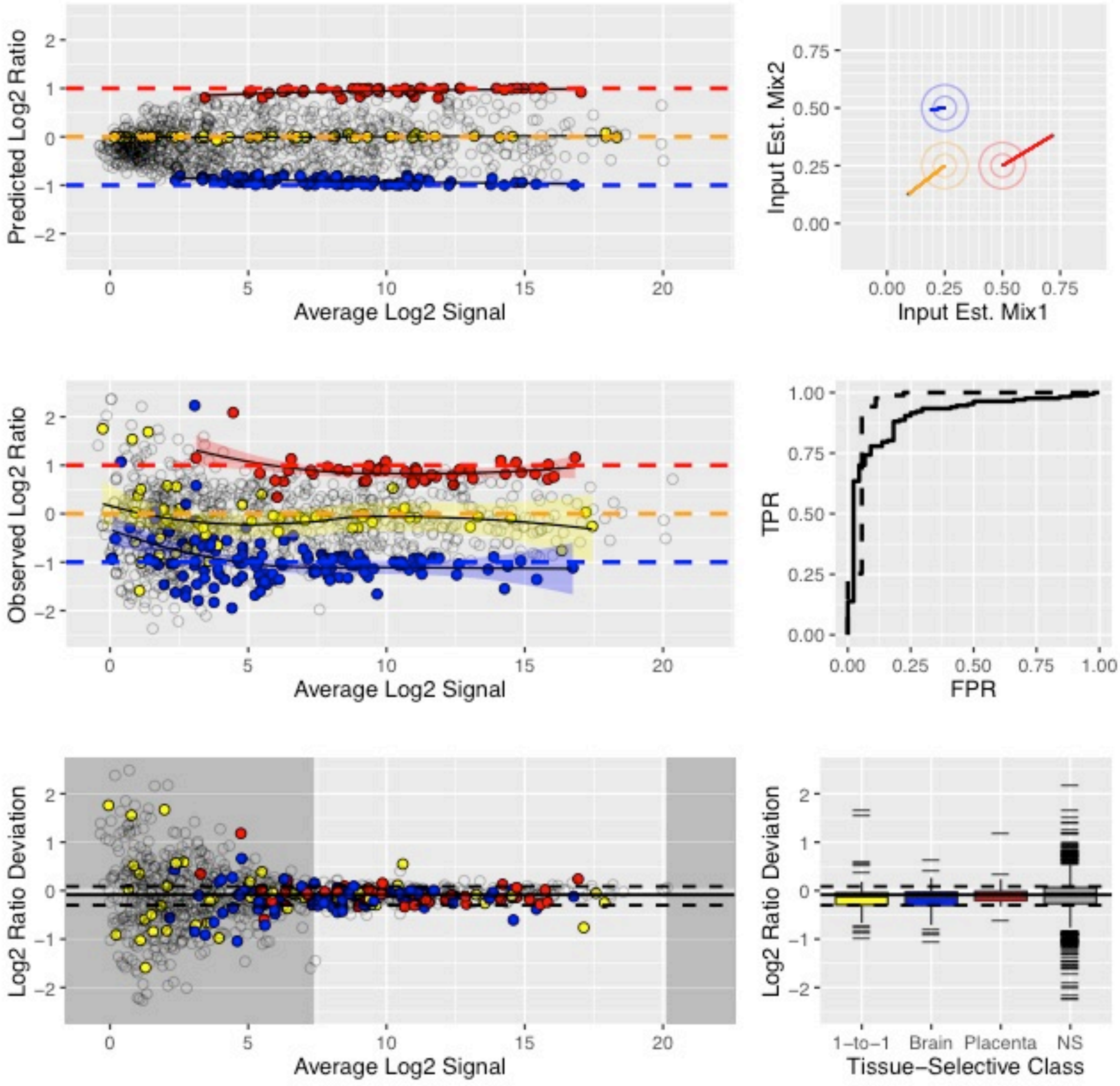

Measurement Process B
Round 4

Class	Detected	Median	IQR	Component	Mix1	Mix2	Bias
1-to-1*	38	−0.0978	0.253	Liver	0.105	0.129	0.189
Brain	102	−0.1303	0.208	Brain	0.246	0.519	0.019
Placenta	58	−0.0977	0.186	Placenta	0.649	0.352	0.181
NS	721	−0.0551	0.402				
All	919	−0.0778	0.328	All			0.388

LowerLimit	Maximum	Range	AUC (All)	AUC (Range)
5.74	18.96	13.23	0.922	0.951

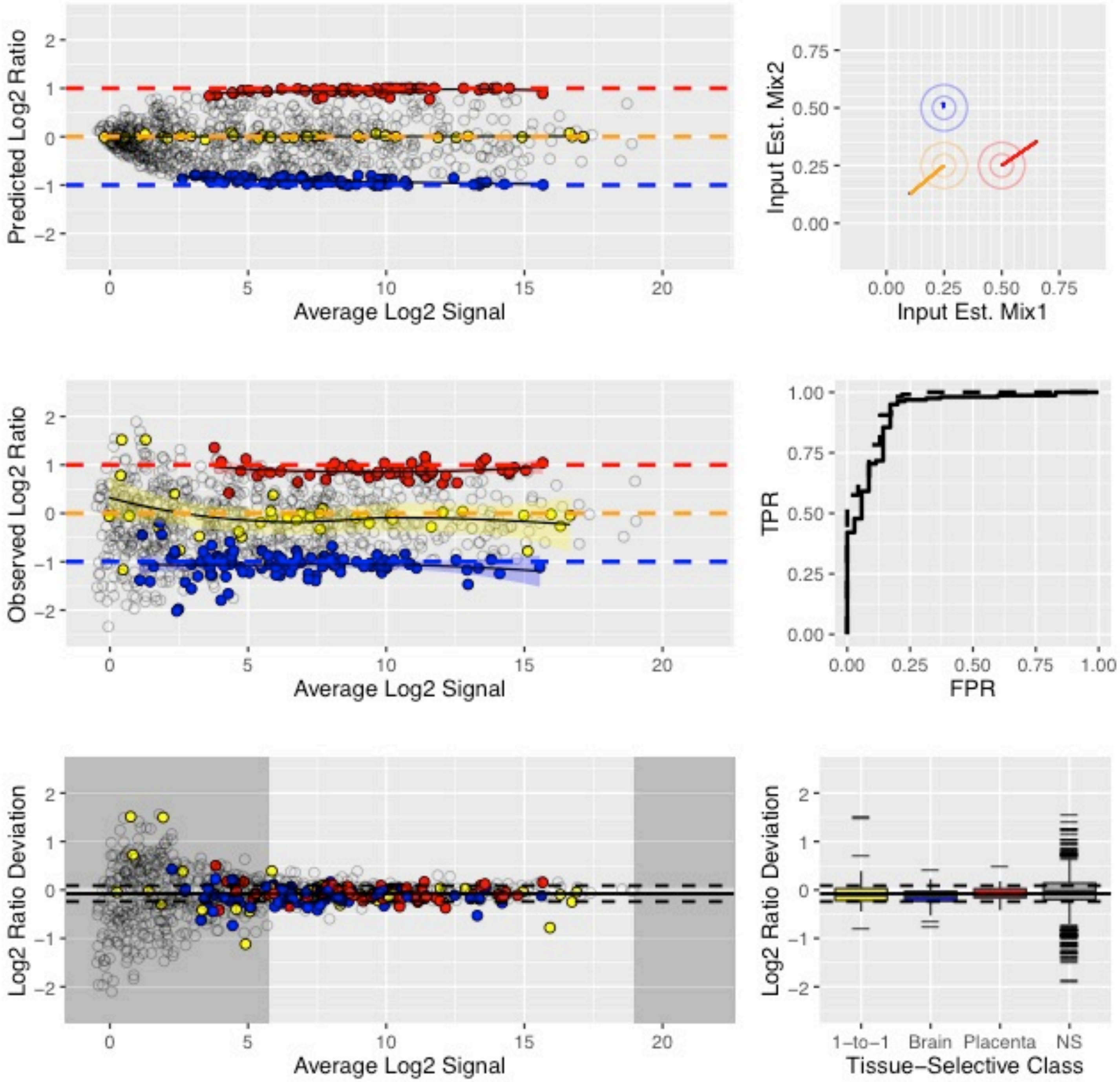

Measurement Process B
Round 5

Class	Detected	Median	IQR	Component	Mix1	Mix2	Bias
1−to−1*	43	−0.0626	0.244	Liver	0.113	0.131	0.181
Brain	78	−0.1009	0.197	Brain	0.236	0.499	0.014
Placenta	44	−0.0525	0.188	Placenta	0.651	0.37	0.193
NS	641	−0.0398	0.339				
All	806	−0.0522	0.322	All			0.389

LowerLimit	Maximum	Range	AUC (All)	AUC (Range)
5.78	19.18	13.4	0.906	0.928

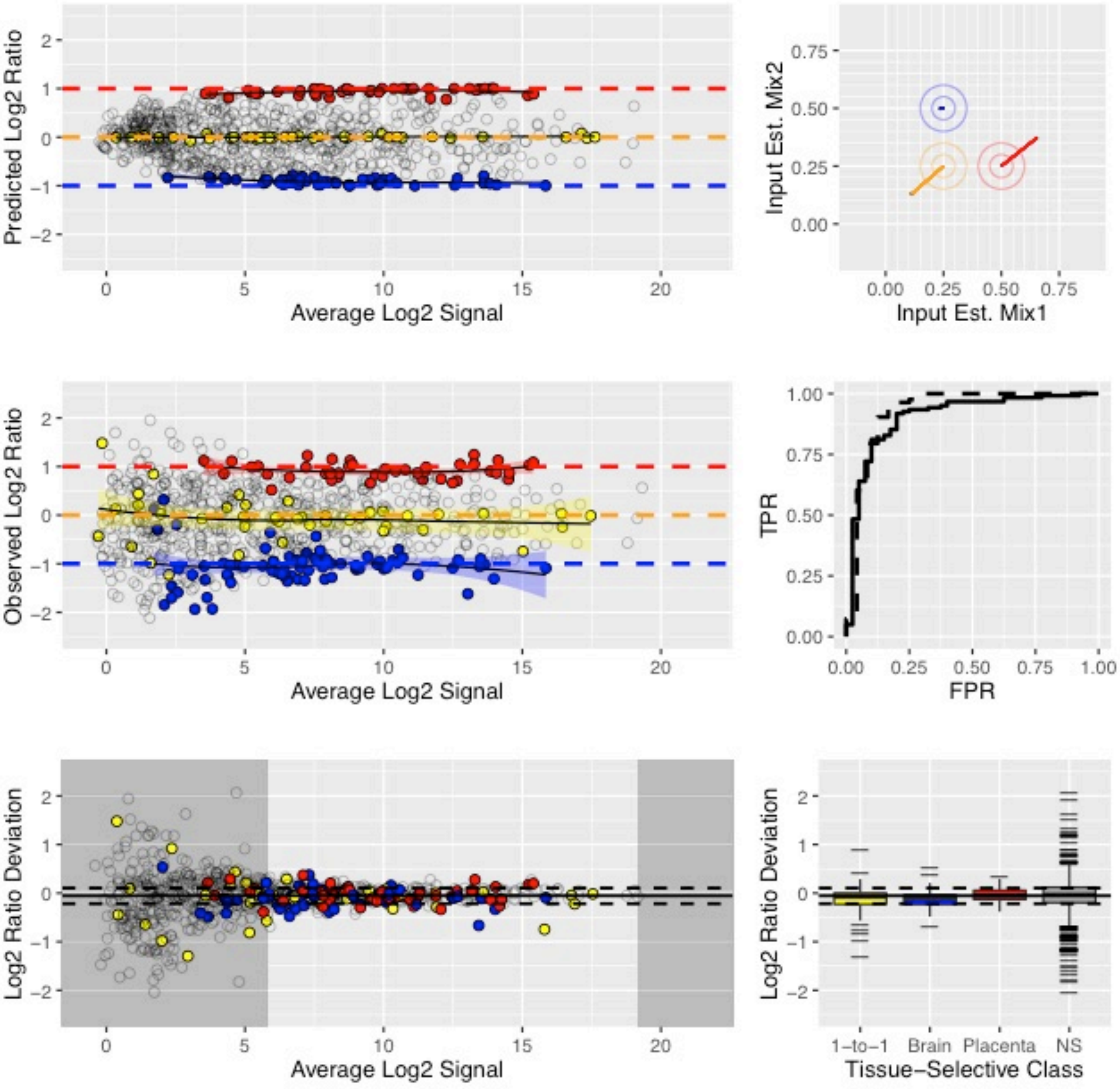

Measurement Process C
Round 3

Class	Detected	Median	IQR	Component	Mix1	Mix2	Bias
1-to-1*	35	0.1137	0.360	Liver	0.132	0.135	0.165
Brain	29	0.1129	0.208	Brain	0.289	0.559	0.071
Placenta	49	0.0566	0.156	Placenta	0.579	0.306	0.097
NS	421	0.0170	0.310				
All	534	0.0297	0.266	All			0.332

LowerLimit	Maximum	Range	AUC (All)	AUC (Range)
5.27	16.27	11	0.944	0.971

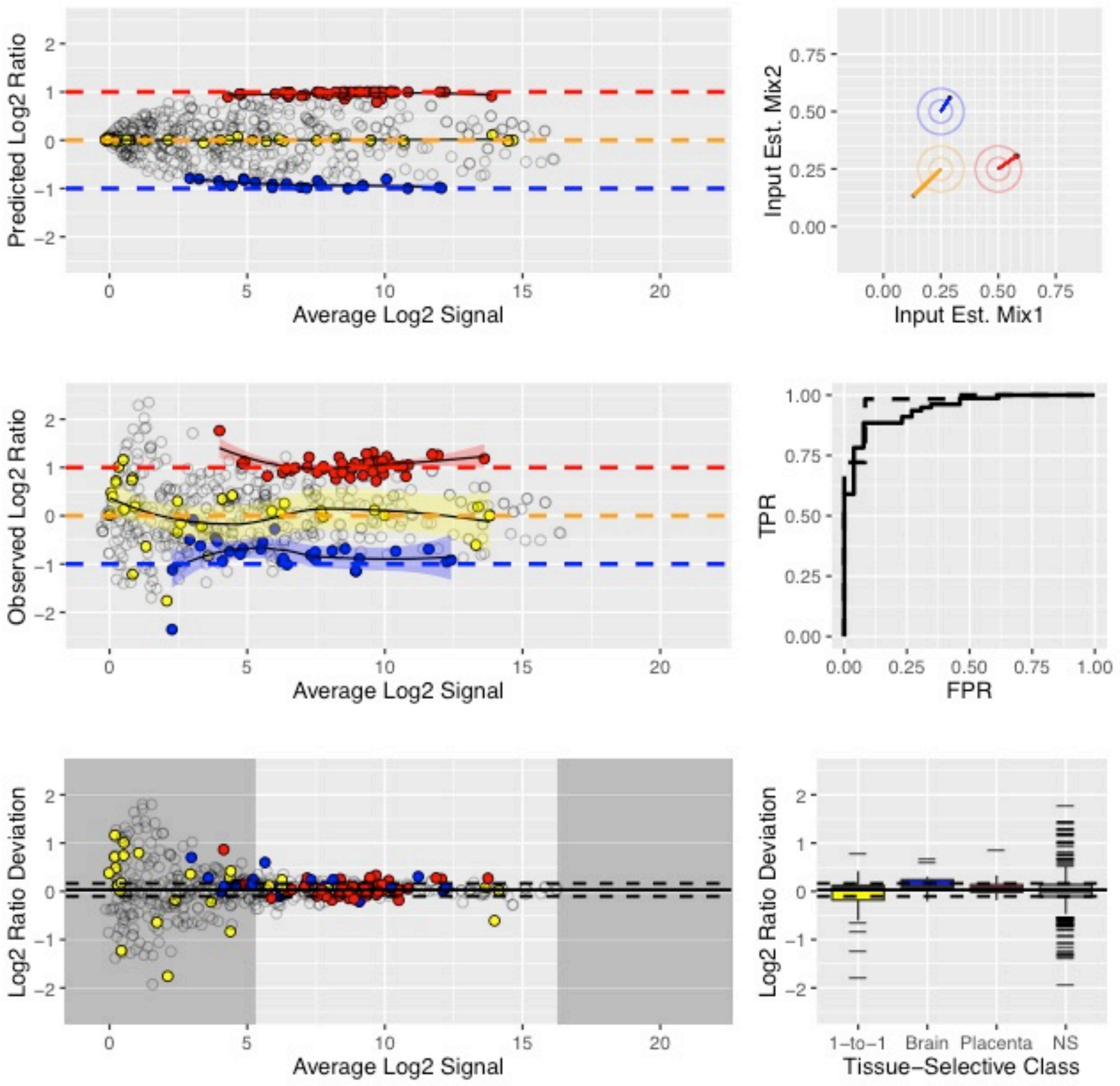

Measurement Process C
Round 4

Class	Detected	Median	IQR	Component	Mix1	Mix2	Bias
1−to−1*	22	−0.0109	0.520	Liver	0.13	0.125	0.173
Brain	16	0.0814	0.120	Brain	0.286	0.587	0.094
Placenta	30	0.2044	0.241	Placenta	0.584	0.287	0.092
NS	277	0.0536	0.661				
All	345	0.0881	0.557	All			0.359

LowerLimit	Maximum	Range	AUC (All)	AUC (Range)
6.27	11.83	5.56	0.831	0.829

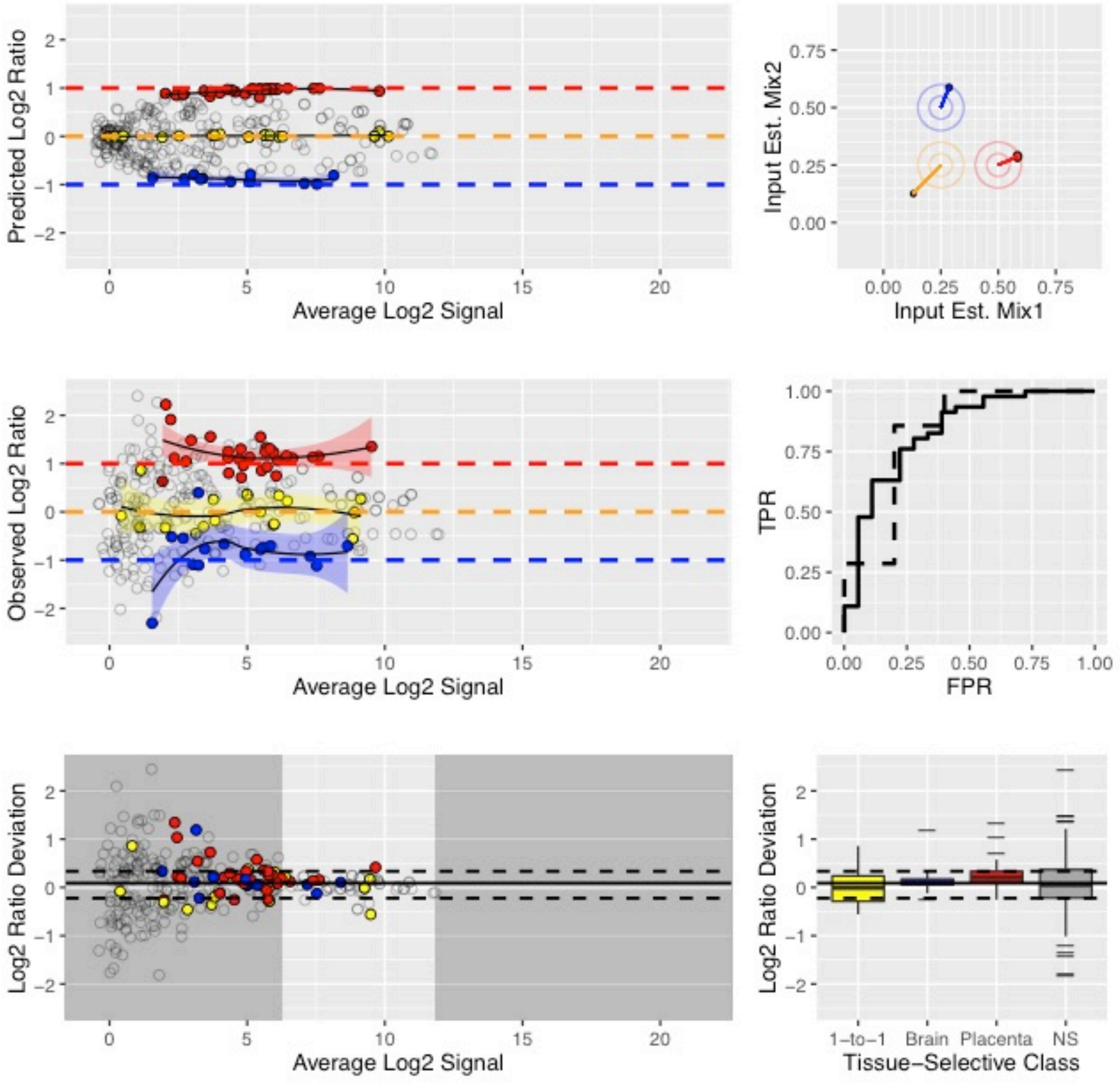

Measurement Process C
Round 5

Class	Detected	Median	IQR	Component	Mix1	Mix2	Bias
1–to–1*	29	0.0123	0.810	Liver	0.087	0.094	0.226
Brain	24	−0.1072	0.502	Brain	0.292	0.621	0.128
Placenta	31	0.1055	0.321	Placenta	0.621	0.285	0.126
NS	433	−0.1493	0.969				
All	517	−0.1040	0.875	All			0.48

LowerLimit	Maximum	Range	AUC (All)	AUC (Range)
10.89	12.43	1.54	0.853	NA

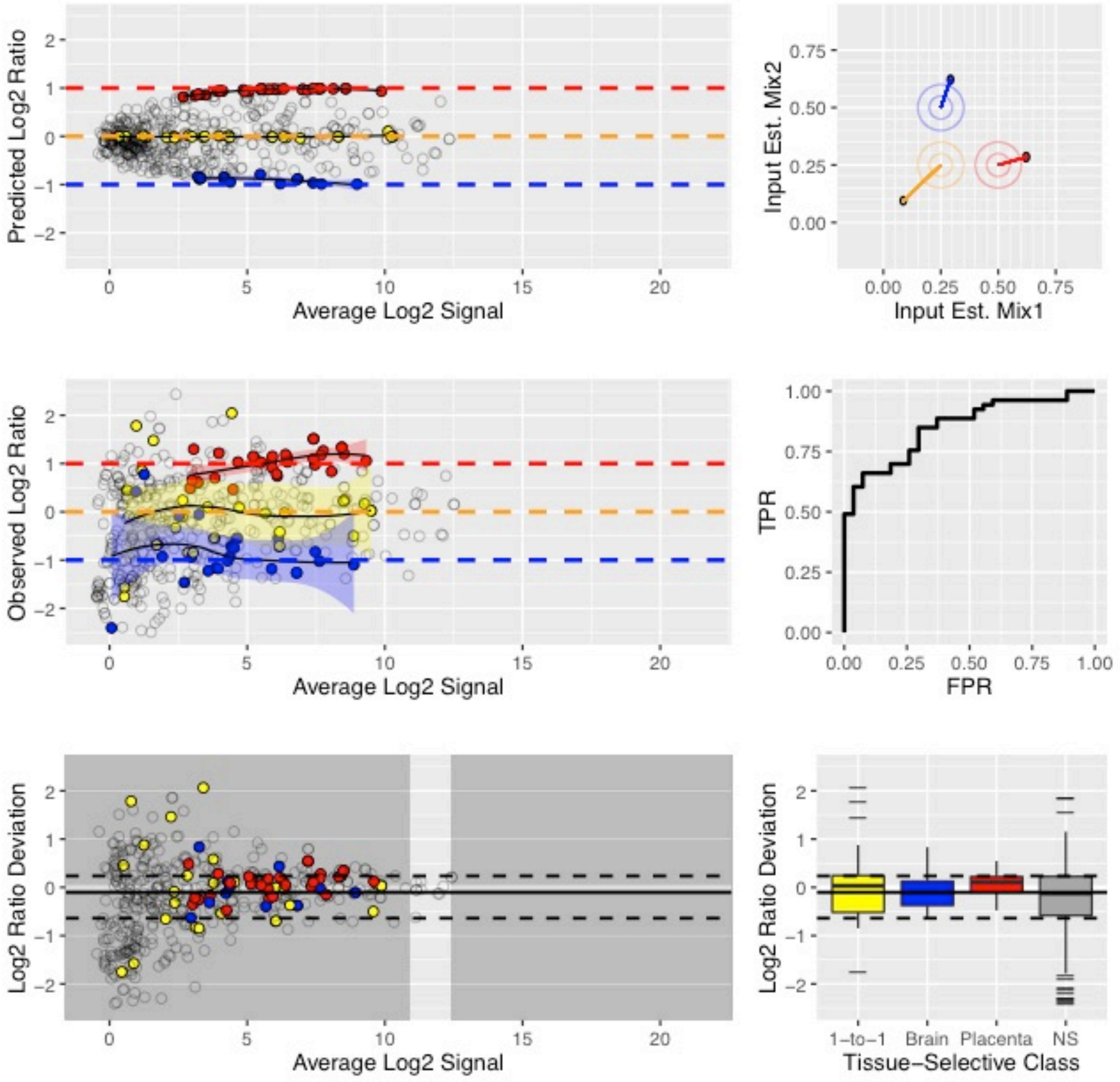

Measurement Process D
Round 3

Class	Detected	Median	IQR	Component	Mix1	Mix2	Bias
1–to–1*	16	−0.5420	0.935	Liver	0.029	0.118	0.258
Brain	18	−0.0767	0.685	Brain	0.14	0.406	0.144
Placenta	9	−0.0019	0.548	Placenta	0.831	0.476	0.401
NS	248	−0.1065	0.627				
All	291	−0.1235	0.627	All			0.803

LowerLimit	Maximum	Range	AUC (All)	AUC (Range)
8.58	10.57	1.99	0.781	NA

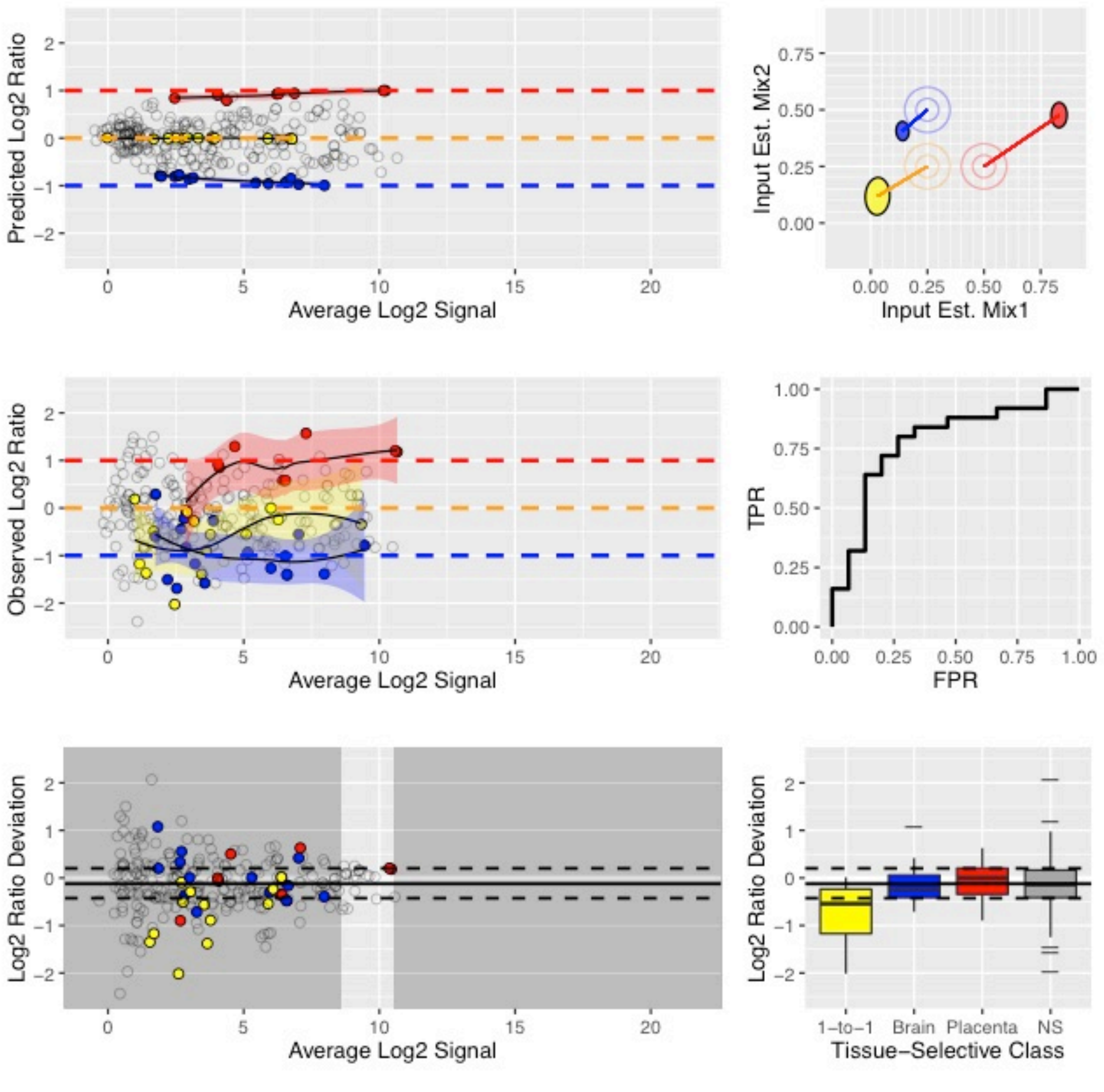

Measurement Process D
Round 4

Class	Detected	Median	IQR	Component	Mix1	Mix2	Bias
1−to−1*	13	−0.0623	0.485	Liver	0.127	0.165	0.149
Brain	13	−0.2649	0.430	Brain	0.208	0.469	0.052
Placenta	2	−0.2309	0.122	Placenta	0.664	0.366	0.201
NS	290	−0.1203	0.540				
All	318	−0.1342	0.534	All			0.402

LowerLimit	Maximum	Range	AUC (All)	AUC (Range)
6.64	10.63	3.99	0.956	NA

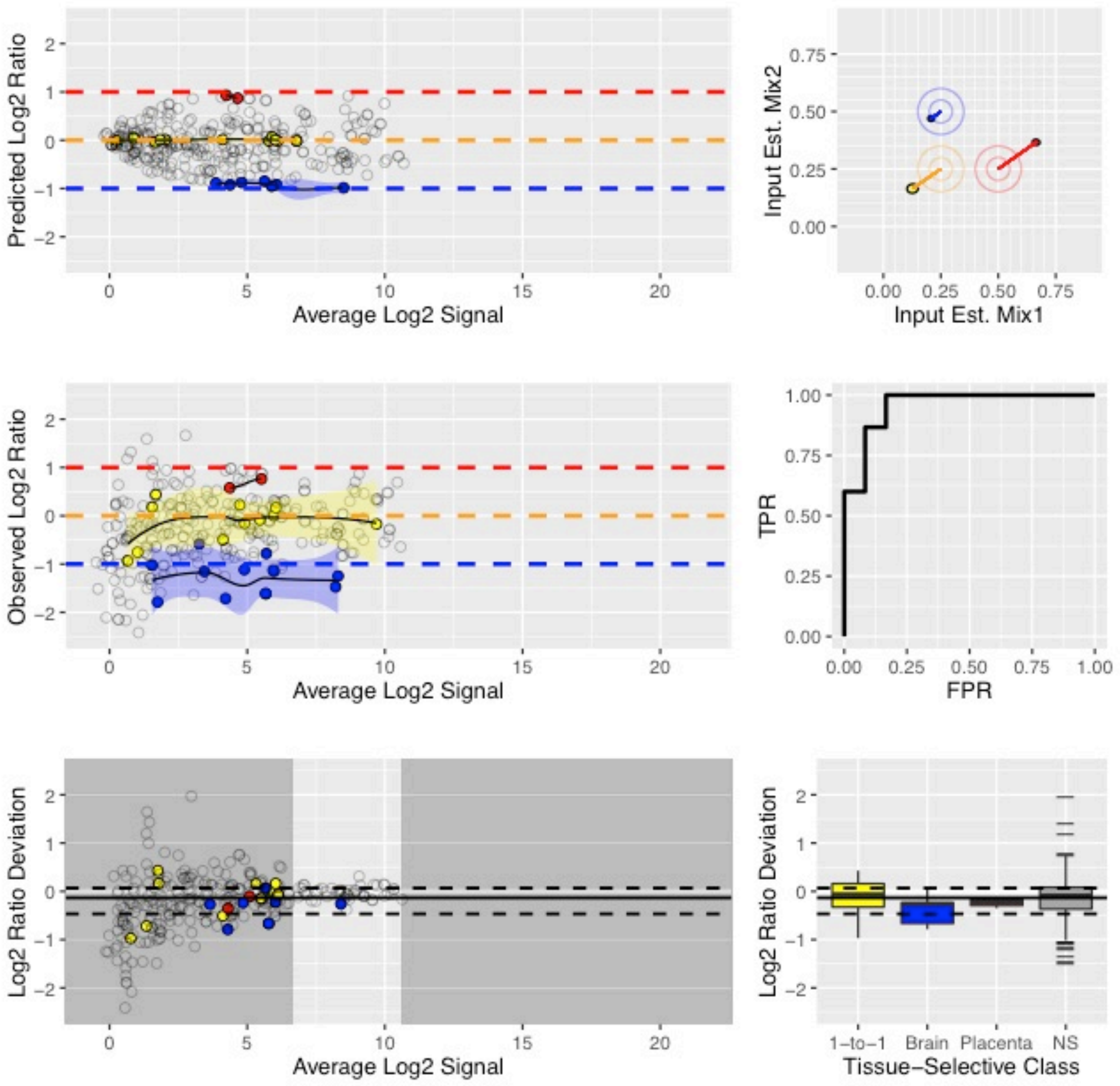

Measurement Process E
Round 3

Class	Detected	Median	IQR	Component	Mix1	Mix2	Bias
1-to-1*	65	−0.0531	0.400	Liver	0.163	0.177	0.114
Brain	8	−0.0674	0.230	Brain	0.156	0.432	0.116
Placenta	24	−0.1022	0.163	Placenta	0.682	0.391	0.23
NS	693	−0.0733	0.397				
All	790	−0.0748	0.392	All			0.46

LowerLimit	Maximum	Range	AUC (All)	AUC (Range)
7.12	13.26	6.14	0.947	1

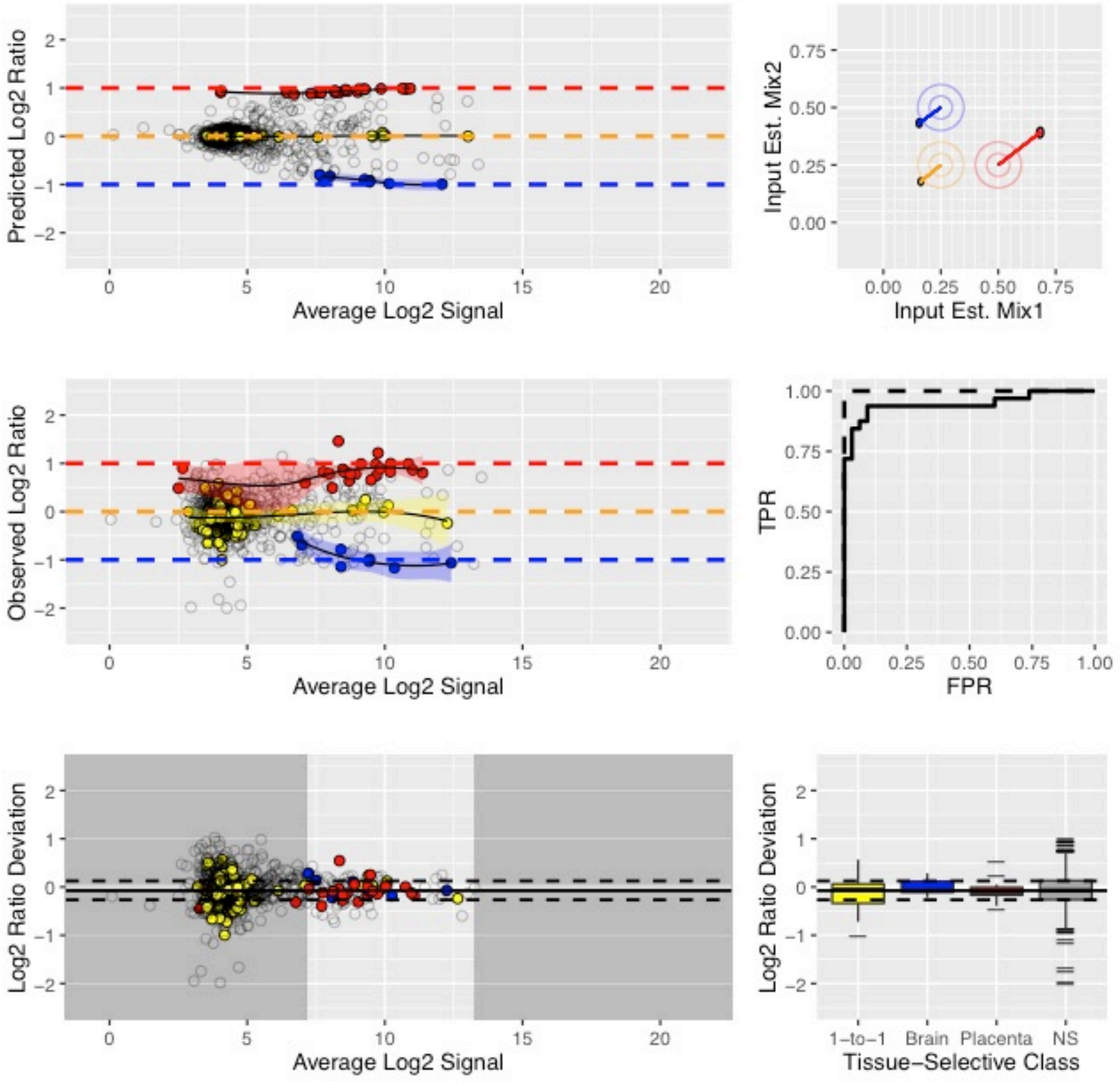

Measurement Process E
Round 4

Class	Detected	Median	IQR	Component	Mix1	Mix2	Bias
1−to−1*	17	0.0183	0.331	Liver	0.13	0.231	0.122
Brain	8	−0.0590	0.116	Brain	0.303	0.441	0.079
Placenta	23	−0.0006	0.070	Placenta	0.567	0.328	0.103
NS	633	−0.1115	0.393				
All	681	−0.0952	0.379	All			0.304

LowerLimit	Maximum	Range	AUC (All)	AUC (Range)
5.6	14.44	8.84	0.985	0.997

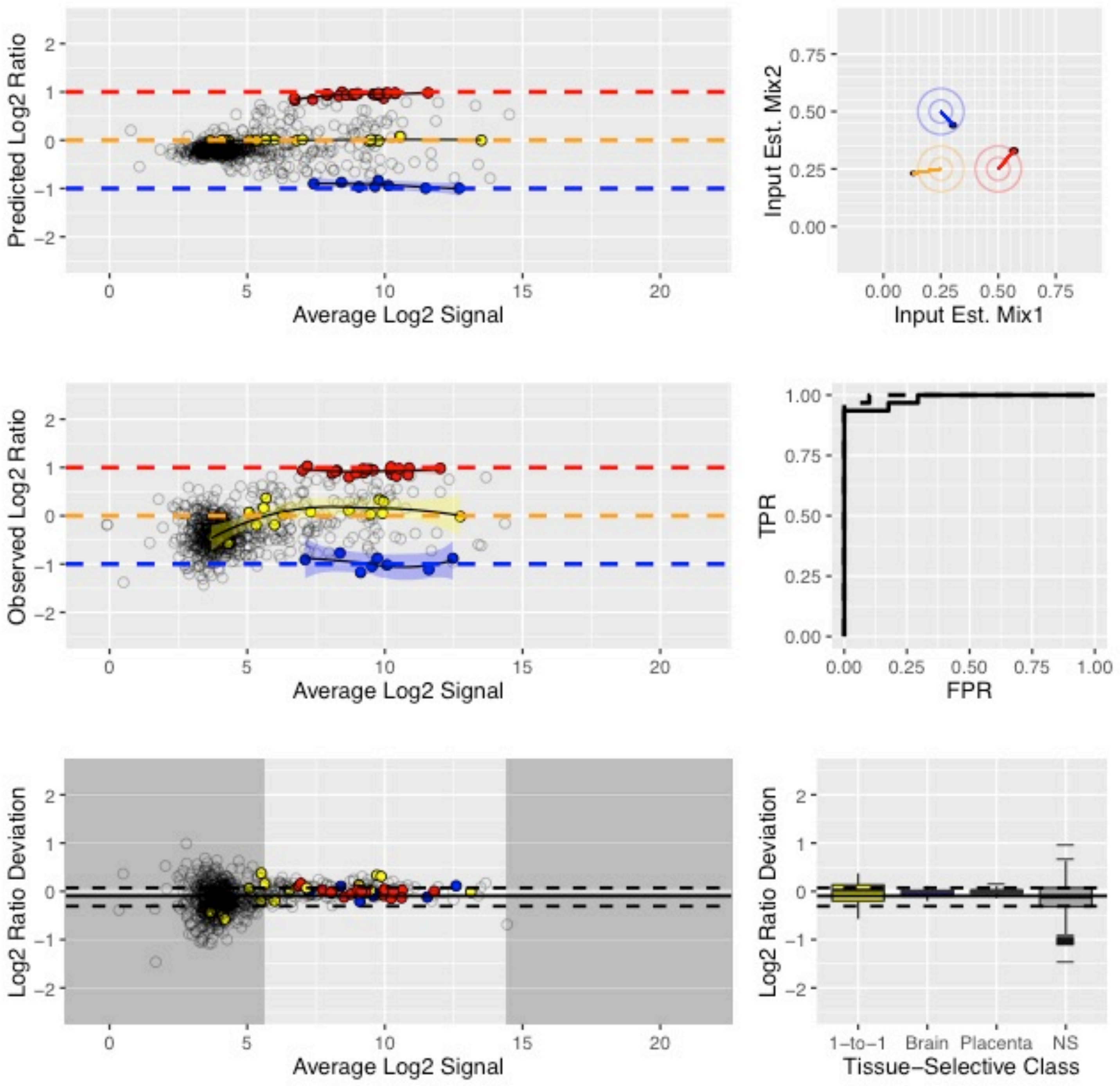

Measurement Process E
Round 5

Class	Detected	Median	IQR	Component	Mix1	Mix2	Bias
1−to−1*	52	−0.3885	0.577	Liver	0.102	0.121	0.197
Brain	16	−0.1919	0.185	Brain	0.202	0.521	0.052
Placenta	37	−0.1918	0.238	Placenta	0.696	0.359	0.224
NS	586	−0.2488	0.643				
All	691	−0.2382	0.599	All			0.473

LowerLimit	Maximum	Range	AUC (All)	AUC (Range)
13.86	15.29	1.43	0.882	1

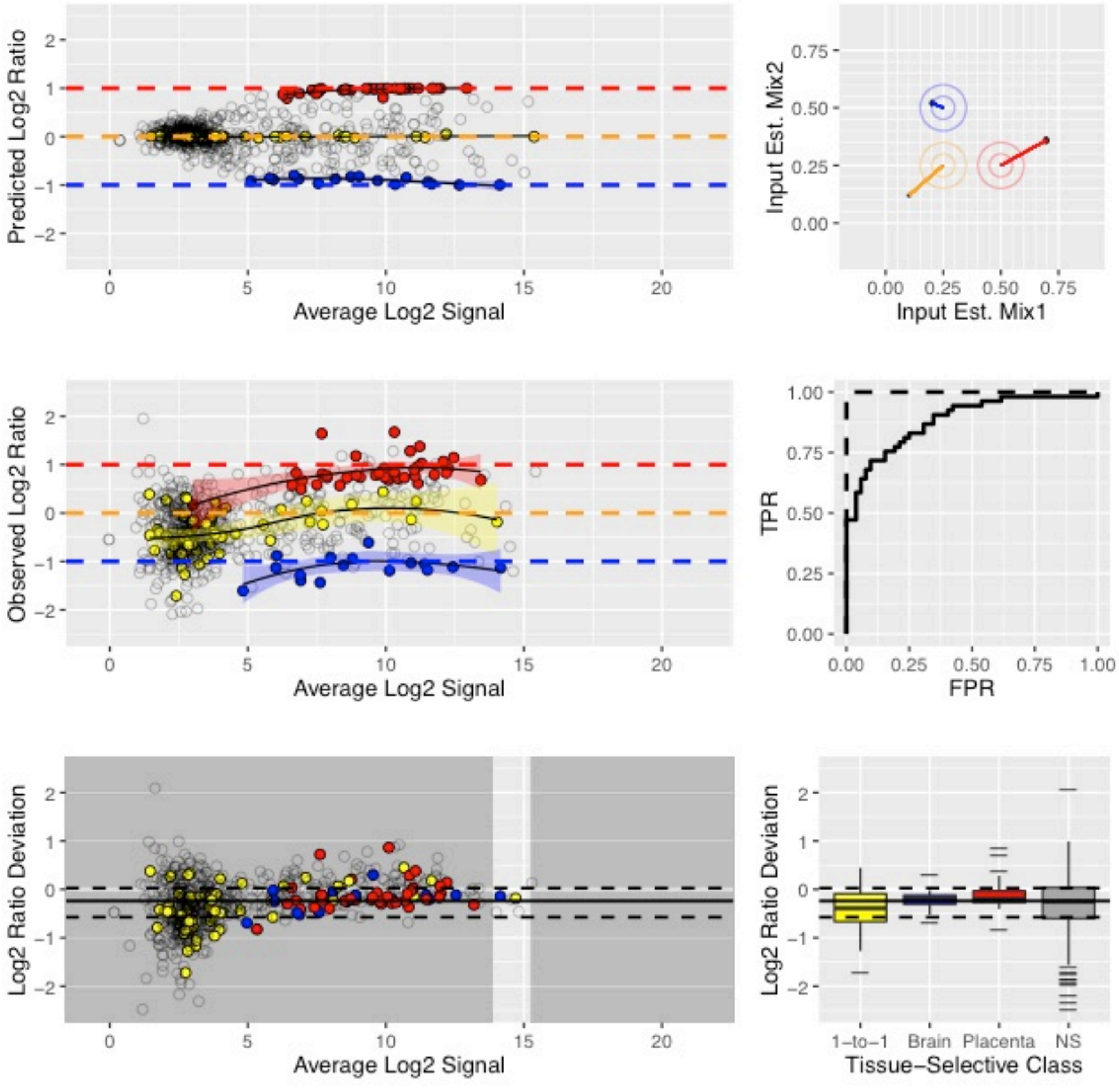

Supplement: Supplementary file 1 — Dashboard views of measurement processes A – E from Rounds 3–5, using three replicates. (PDF 1960 kb) [file 12864_2018_4496_MOESM1_ESM.pdf]
