# Supplementary material for: Summarizing performance for genome scale measurement of miRNA: reference samples and metrics
Source: BMC Genomics. 2018 Mar 6;19:180. doi: 10.1186/s12864-018-4496-1 (PMC5838960; doi:10.1186/s12864-018-4496-1)
Supplement: Supplementary file 2 — Protocols for measurement processes A – F. (PDF 395 kb) [file 12864_2018_4496_MOESM2_ESM.pdf]

Additional File 2

Protocol Description: Measurement Process A

1. **Site ID:** Lab008 **Technology:** NGS **Protocol version:** A

2. **Brief protocol or citation to protocol.**
   New England Biolabs NEBNext Multiplex Small RNA Library Prep Set for Illumina Instruction Manual (using PAGE gel for size selection)

3. **Sample storage conditions (temperature and duration prior to processing).**
   -80 °C, 1 week – 3 months after receipt

4. **Amount of input sample.**
   200 ng

5. **Kits and reagents, including manufacturer and catalog numbers.**
   New England Biolabs - NEBNext Multiplex Small RNA Library Prep Set for Illumina (Set 1), Catalog #E7300S
   New England Biolabs - NEBNext Multiplex Small RNA Library Prep Set for Illumina (Set 2), Catalog # E7580S
   Life Technologies, Inc. - 6% Novex TBE PAGE gel 1.0 mM 10-well, Catalog # EC6265BOX

6. **Instrumentation, including manufacturer and model numbers.**
   Agilent 2100 BioAnalyzer
   Illumina HiSeq 2500

7. **Data analysis and processing software, including manufacturer and version.**
   FASTQ files were trimmed with the fastx_clipper utility and adapter sequence 5'-AGATCGGAAGAGCACACGTCTGAACTCCAGTCAC-3' using the FASTX toolkit (version 0.0.13.2).
   Trimmed reads >= 15 bases in length were aligned to human genome build hg19 using Bowtie (v 1.1.1), allowing up to 3 mismatches per alignment and 10 alignments per read.
   Uniquely-aligning reads were used to obtain microRNA-level expression with the 'coverageBed' utility in the BEDtools software suite (version 2.22.1) and a BED file of 2,794 mature microRNA loci from miRBase (version 20).

8. **Note any changes in the methods that occurred between rounds.**
   Sequencing libraries from 9 Round #2 RNA samples were pooled and sequenced in one lane of a High-Output Single-Read flow cell on HiSeq 2500. For Round #3, #4, and #5, each round had 15 RNA samples and sequencing libraries of each round were pooled and sequenced on a Rapid Single-Read flow cell.

Protocol Description: Measurement Process B

1. **Site ID:** Lab007 **Technology:** NGS **Protocol version:** A

2. **Brief protocol or citation to protocol.**
The samples were thawed on ice and 1 µg of RNA was moved forward into small RNA sample preparations. The Illumina small RNA TruSeq kit (RS-200-0048; Illumina) was used for preparing all the samples for sequencing. Kit reagents were used in a half reaction (Burgos KL, et al. (2013) RNA 19:712–22). A total of 12 cycles were preformed for PCR and each sample was assigned one of 48 possible indices. Indexed samples were run on a gel and purified away from the adaptor band. Samples were quantified with the Agilent High Sensitivity DNA Kit (5067-4626; Agilent). The samples were then pooled and placed on a single read Illumina V3 flowcell (GD-401-3001; Illumina). The flowcells ran on the Illumina HiSeq sequencing platform (HiSeq 2500; Illumina).

3. **Sample storage conditions (temperature and duration prior to processing).**
Stored at −80 °C, for up to 3 months

4. **Amount of input sample.**
1 µg

5. **Kits and reagents, including manufacturer and catalog numbers.**
Illumina small RNA TruSeq kit (RS-200-0048; Illumina);
Agilent High Sensitivity DNA Kit (5067-4626; Agilent);
single read Illumina V3 flowcell (GD-401-3001; Illumina)

6. **Instrumentation, including manufacturer and model numbers.**
Illumina HiSeq 2500

7. **Data analysis and processing software, including manufacturer and version.**
Raw FASTQ files were generated and demultiplexed using the Illumina CASAVA v1.8.4 pipeline. Adapters were trimmed with cutadapt v1.8.3, with a quality score cutoff of 30 and a minimum length of 14 nt. Samples were then aligned with sRNABench, which facilitates read alignment to various other RNA libraries with hierarchical mapping using bowtie v2.1.0. Reads mapping to human rRNA (ribosomal RNA from NCBI) were removed and then the remaining reads were mapped to the miRNA database (miRBase v21), with a seed length of 19 nt and 1 mismatch allowed.

8. **Note any changes in the methods that occurred between rounds.**
None

Protocol Description: Measurement Process C

1. **Site ID:** Lab006 **Technology:** NGS **Protocol version:** B

2. **Brief protocol or citation to protocol.**
   Illumina TruSeq Small RNA Library Prep Kit Reference Guide

3. **Sample storage conditions (temperature and duration prior to processing).**
   Samples sets prepared at NIST: 07/02/14
   Round 3: ~12 weeks in storage at −80 °C (run completed 09/23/14)
   Round 4: ~44 weeks in storage at −80 °C (run completed 05/07/15)
   Round 5: ~90 weeks in storage at −80 °C (run completed 03/22/16)

4. **Amount of input sample.**
   1 µg

5. **Kits and reagents, including manufacturer and catalog numbers.**
   Illumina
   TruSeq Small RNA Sample Preparation Kit – Set A (24 rxns; indices 1-12)
   Catalog # RS-200-0012
   TruSeq Small RNA Sample Preparation Kit – Set B (24 rxns; indices 13-24)
   Catalog # RS-200-0024
   Sage Science
   3 % Agarose Gel Cassettes for Blue Pippin (size selection)
   Catalog # BDF3010

6. **Instrumentation, including manufacturer and model numbers.**
   Agilent 2100 BioAnalyzer
   Sage Science Blue Pippin
   Illumina MiSeq

7. **Data analysis and processing software, including manufacturer and version.**
   Filtered reads for quality using fastq quality filter
   Trimmed adapters using fastx clipper
   Mapped reads to miRBase v20 using SHRiMP
   Counted output number of reads mapped to miR with a custom Perl script

8. **Note any changes in the methods that occurred between rounds.**
   No changes

Protocol Description: Measurement Process D

1. **Site ID:** Lab006 **Technology:** NGS **Protocol version:** A

2. **Brief protocol or citation to protocol.**
   Ion Total RNA-Seq Kit V2 User Guide

3. **Sample storage conditions (temperature and duration prior to processing).**
   Samples sets prepared at NIST: 07/02/14
   Round 3: ~07 weeks in storage at −80 °C (run completed 08/21/14)
   Round 4: ~68 weeks in storage at −80 °C (run completed 10/22/15)
   Round 5: not done

4. **Amount of input sample.**
   1 μg

5. **Kits and reagents, including manufacturer and catalog numbers.**
   Ion Total RNA-Seq v2
   Catalog # 4475936
   Ion Xpress RNA-Seq Barcode 1-16 Kit
   Catalog # 4475485

6. **Instrumentation, including manufacturer and model numbers.**
   Agilent 2100 BioAnalyzer
   IonTorrent PGM

7. **Data analysis and processing software, including manufacturer and version.**
   Filtered reads for quality using fastq quality filter
   Trimmed adapters using fastx clipper
   Mapped reads to miRBase v20 using SHRiMP
   Counted output number of reads mapped to miR with a custom Perl script

8. **Note any changes in the methods that occurred between rounds.**
   No changes

Protocol Description: Measurement Process E

1. **Site ID:** Lab005 **Technology:** HYB **Protocol version:** A

2. **Brief protocol or citation to protocol.**
   Digital multiplexed NanoString nCounter analysis system was performed. RNA samples were analyzed by nCounter Human miRNA panels (800 human miRNA targets) with 5 internal housekeeping controls and 5 Spike-in controls. nCounter miRNA Expression Assay kits were used to analyze the digital detection of the miRNAs in a single reaction. Digital detection was performed in two parts; (a) Transcripts detected by probes bound to complimentary segments of DNA which are attached to a unique string of colored fluorophores. (b) Number of total transcripts in the sample was counted by the number of times is the fluorophore was detected; scanning was performed in 555 fields of view. Nanostring counts represent molecules/100 ng of total RNA. Following hybridization, counts were analyzed by the nCounter Digital Analyzer. The resulting raw data were imported into nSolver for the analysis.

3. **Sample storage conditions (temperature and duration prior to processing).**
   −80 °C, < 1 month after receipt

4. **Amount of input sample.**
   100 ng

5. **Kits and reagents, including manufacturer and catalog numbers.**
   HSA miRNA v2/v2.1 Assay Kit 12 rxn GXA-MIR2-12

6. **Instrumentation, including manufacturer and model numbers.**
   Nanostring n-Counter Platform- Nanostring Technologies

7. **Data analysis and processing software, including manufacturer and version.**
   nSolver Software

8. **Note any changes in the methods that occurred between rounds.**
   No deviations in kit protocols
   No changes in default instrument settings
   No changes in the lots used per round

Protocol Description: Measurement Process F

1. **Site ID:** Lab004 **Technology:** PCR **Protocol version:** A

2. **Brief protocol or citation to protocol.**
   Applied Biosystems "Protocol for running custom RT and preamplification pools on custom TaqMan array microRNA Cards" (Publication PN 4478705 Revision date: January 2013) using sub-protocol for Custom TaqMan Array MicroRNA Cards without pre-amplification.

3. **Sample storage conditions (temperature and duration prior to processing).**
   −80 °C, < 1 month after receipt

4. **Amount of input sample.**
   1 ug

5. **Kits and reagents, including manufacturer and catalog numbers.**
   Custom 384-well TaqCards for 32 TaqMan miRNA assays (listed below), including custom RT primers. Three replicate wells per assay per sample. Four samples per TaqCard.
   TaqMan MicroRNA Reverse Transcription Kit (PN 4366596) for RT and TaqMan Universal Master Mix II, No AmpErase UNG for PCR.

6. **Instrumentation, including manufacturer and model numbers.**
   Applied Biosystems ViiA7 real-time PCR instrument

7. **Data analysis and processing software, including manufacturer and version.**
   Applied Biosystems ViiA™ 7 software

8. **Note any changes in the methods that occurred between rounds.**
   No deviations in kit protocols
   No changes in default instrument settings
   Different reagent lots used per round

Gene Symbols (Assay ID): hsa-miR-10a (387); hsa-miR-150 (473); hsa-miR-23a (399); hsa-miR-369-5p (1021); hsa-miR-125a-5p (2198); hsa-miR-152 (475); hsa-miR-26b (407); hsa-miR-375 (564); hsa-miR-128a (2216); hsa-miR-183 (2269); hsa-miR-323-3p (2227); hsa-miR-382 (572); U6 snRNA (1973); mmu-miR-187 (1193); hsa-miR-335 (546); mmu-miR-451 (1141); mmu-miR-129-3p (1184); hsa-miR-191 (2299); hsa-miR-340 (2258); mmu-miR-495 (1663); hsa-miR-129 (590); hsa-miR-195 (494); hsa-miR-342-3p (2260); hsa-miR-9 (583); hsa-miR-132 (457); hsa-miR-204 (508); hsa-miR-361 (554); hsa-miR-98 (577); mmu-miR-137 (1129); hsa-miR-218 (521); hsa-miR-365 (1020); hsa-miR-192 (491)

Protocol Description: Measurement Process F

1. **Site ID:** Lab001 **Technology:** PCR **Protocol version:** A

2. **Brief protocol or citation to protocol.**
   Applied Biosystems "TaqMan® Small RNA Assays" (Publication 4364031 Rev. E) using the following custom sub-protocol for multiplexing the RT reactions:
   1) The RNA samples were diluted to 2 ng/µl.
   2) The composition of the resulting reactions was as follows:

RNA (2 ng/µl)	5	µl
H_2O	0	µl
(note: water substituted with two additional RT primers)		
dNTPs (100 µM)	0.15	µl
10X buffer	1.5	µl
RNase inhibitor	0.19	µl
RT primers* (each)	2.39	µl
MultiScribe RT	1	µl

   3) Mastermix without the RNA template was prepared for each of the group.
   10 µl of the mastermix was added to the PCR plate wells, and
   5 µl of 2 ng/µl RNA was added and mixed by pipetting.
   All reactions were performed in triplicates.

3. **Sample storage conditions (temperature and duration prior to processing).**
   −80 °C, < 1 month after receipt

4. **Amount of input sample.**
   10 ng

5. **Kits and reagents, including manufacturer and catalog numbers.**
   96-well plates (Bio-Rad, PN HSS9601)
   Plate microseal (Bio-Rad, PN MSB1001)
   TaqMan MicroRNA assays (Life Technology, PN 4427975)
   TaqMan MicroRNA Reverse Transcription Kit (PN 4366596).

6. **Instrumentation, including manufacturer and model numbers.**
   Bio-Rad iCycler real-time PCR instrument

7. **Data analysis and processing software, including manufacturer and version.**
   Bio-Rad MyiQ v1.0 software

8. **Note any changes in the methods that occurred between rounds.**
   Different reagent lots used per round

Gene Symbols (Assay ID):
*Group 1: hsa-miR-451 (1141), hsa-miR-375 (564), and hsa-miR-335-5p (546)
Group 2: hsa-miR-218-5p (521), hsa-miR-125b-5p (449), and hsa-miR-19b-3p (396)

Protocol Description: Measurement Process F

1. **Site ID:** Lab003 **Technology:** PCR **Protocol version:** A

2. **Brief protocol or citation to protocol.**
   Applied Biosystems "TaqMan® Small RNA Assays" (Publication 4364031)

3. **Sample storage conditions (temperature and duration prior to processing).**
   Round 3: Stored in −80 °C freezer, Received 07/08/14, Tested 08/27/14
   Round 4: Stored in −80 °C freezer, Received 01/21/15, Tested 07/05/15
   Round 5: Stored in −80 °C freezer, Received 07/15/15, Tested 07/25/15

4. **Amount of input sample.**
   50 ng per reaction

5. **Kits and reagents, including manufacturer and catalog numbers.**
   TaqMan MicroRNA Reverse Transcriptase Kit, Applied Biosystems #4366596
   Water, DEPC-treated, RNAse Free, Ambion by Life Technologies #AM9916
   TaqMan 2X Universal PCR Master Mix, Applied Biosystems #4364341
   TaqMan MicroRNA Assays, Applied Biosystems #4427975

6. **Instrumentation, including manufacturer and model numbers.**
   Applied Biosystems GeneAmp 9700
   Bio-Rad CFX96 Touch Real-Time PCR Detection System

7. **Data analysis and processing software, including manufacturer and version.**
   Bio-Rad CFX Manager Version 3.1

8. **Note any changes in the methods that occurred between rounds.**
   DEPC-treated water supplier was changed between Rounds 3 and 4
   Three miRNA assays were added in Rounds 4 and 5

Gene Symbols (Assay ID):
Rounds 3 – 5: hsa-miR-375 (564) and hsa-miR-451a (1141)
Rounds 4 – 5: hsa-miR-125b-5p (449), hsa-miR-218-5p (521), and hsa-miR-335-5p (546)
