# Supplementary material for: Summarizing performance for genome scale measurement of miRNA: reference samples and metrics
Source: BMC Genomics. 2018 Mar 6;19:180. doi: 10.1186/s12864-018-4496-1 (PMC5838960; doi:10.1186/s12864-018-4496-1)
Supplement: Supplementary file 3 — Instructions for using measurement assurance pipeline online. (PDF 223 kb) [file 12864_2018_4496_MOESM3_ESM.pdf]

Instructions for Accessing the miRNA Analysis Pipeline for the Genome-scale Measurement Assurance Project of the EDRN

(see **Figure S1** for overview)

1. From the EDRN Public Portal navigate to LabCAS login page:
    a. Select "Informatics" from sidebar
    b. Select "LabCAS" from Tools list
    c. Log in

    Opens Manage Workflows page

2. From the Manage Workflows page (skip to Step 5, to view existing datasets)
    a. Select "Upload Files"
    b. Select "NIST Dataset"

    Opens Upload Metadata page

3. From the Upload Metadata page:
    a. Confirm "Lab ID"
    b. Select the "Protocol ID" or select "New Protocol"
        i. If new, see "Directions for preparing protocol descriptions" and refer to **Figure S2**.
    c. Select the "Sample ID" or select "New Sample"
        i. If new, see "Directions for preparing protocol descriptions" and refer to **Figure S3**.
    d. Enter data collection date

    Opens Ready for Files page

4. From the Ready for Files page:
    a. Confirm metadata to be linked to dataset
    b. Select dataset to be uploaded (refer to **Figure S4** for format):
        i. *browse* to the correct filename on the participants computer, or
        ii. *drag* the correct filename onto the webpage
    c. Select "Start Uploading"
    d. Select "Finish"

    Generates PDF of dashboard view using default parameters
    Returns to Manage Workflows page (step 2)

5. From Manage Workflows page:
    a. Select "NIST Product" to view uploaded datasets
    b. Select a dataset from the collection *(LabID_ProtocolID_SampleID)*
    c. Select file(s) for download:
        i. (*.txt) text/plain for off-line analysis
        ii. (*.pdf) application/pdf to view dashboard results

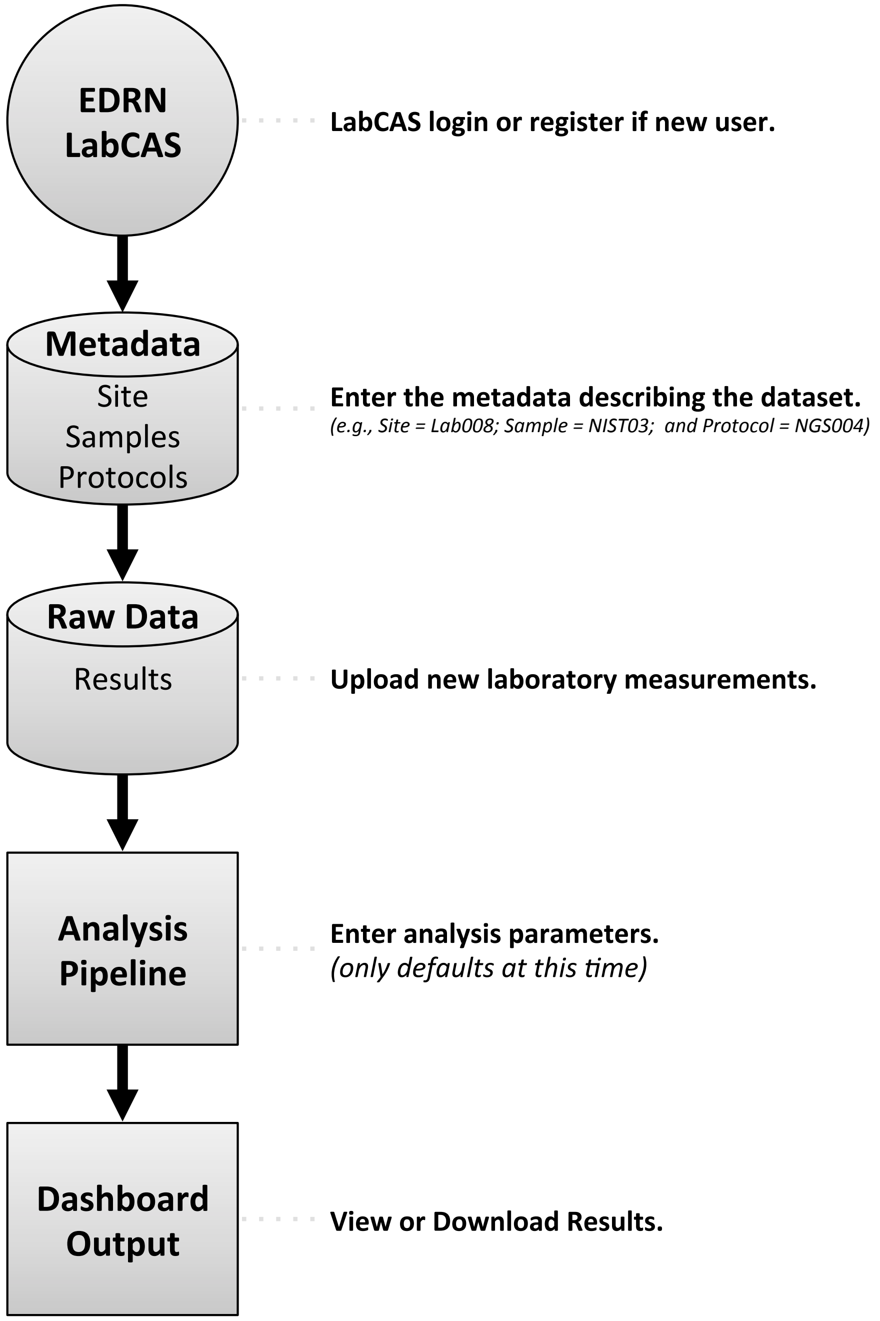

Figure S1. Workflow for uploading and analyzing miRNA benchmarking samples using the EDRN Genome-Scale Measurement Assurance portal.

Directions for preparing protocol descriptions:
If your laboratory is entering a dataset that was derived from a protocol different from those listed (Step 3b), download the protocol closest to the one used and edit it accordingly (see **Figure S2** for example). If no similar protocol has been described previously, use the template below to prepare a new protocol description.

Protocol Description

1. SiteID: *(replace with LabCAS LabID)*

   Technology: [ ] HYB [ ] NGS [ ] PCR [ ] other
   If other, describe:

   Protocol version: *(use "A" for the first protocol submitted, incrementally letter additional protocols from your site)*

2. Brief protocol or citation to protocol.
   (limited to < 1,000 characters)

3. Sample storage conditions.
   (temperature and duration prior to processing)

4. Amount of input sample.
   (ng or µg)

5. Kits and reagents.
   (including manufacturer and catalog numbers)

6. Instrumentation
   (including manufacturer and model numbers)

7. Data analysis and preprocessing software
   (including manufacturer and version)

8. Note any changes in the methods that occurred between runs

Send new or modified protocols to contact information located at:
https://edrn.nci.nih.gov/microrna.
A corresponding ProtocolID will be added to the analysis pipeline.

Protocol Description

1. **Site ID:** Lab008 **Technology:** NGS **Protocol version:** A

2. **Brief protocol or citation to protocol.**
New England Biolabs NEBNext Multiplex Small RNA Library Prep Set for Illumina Instruction Manual (using PAGE gel for size selection)

3. **Sample storage conditions (temperature and duration prior to processing).**
-80 °C, 1 week – 3 months after receipt

4. **Amount of input sample.**
200 ng

5. **Kits and reagents, including manufacturer and catalog numbers.**
New England Biolabs - NEBNext Multiplex Small RNA Library Prep Set for Illumina (Set 1), Catalog #E7300S
New England Biolabs - NEBNext Multiplex Small RNA Library Prep Set for Illumina (Set 2), Catalog # E7580S
Life Technologies, Inc. - 6% Novex TBE PAGE gel 1.0 mM 10-well, Catalog # EC6265BOX

6. **Instrumentation, including manufacturer and model numbers.**
Agilent 2100 BioAnalyzer
Illumina HiSeq 2500

7. **Data analysis and processing software, including manufacturer and version.**
FASTQ files were trimmed with the fastx_clipper utility and adapter sequence 5'-AGATCGGAAGAGCACACGTCTGAACTCCAGTCAC-3' using the FASTX toolkit (version 0.0.13.2).
Trimmed reads >= 15 bases in length were aligned to human genome build hg19 using Bowtie (v 1.1.1), allowing up to 3 mismatches per alignment and 10 alignments per read.
Uniquely-aligning reads were used to obtain microRNA-level expression with the 'coverageBed' utility in the BEDtools software suite (version 2.22.1) and a BED file of 2,794 mature microRNA loci from miRBase (version 20).

8. **Note any changes in the methods that occurred between rounds.**
Sequencing libraries from 9 Round #2 RNA samples were pooled and sequenced in one lane of a High-Output Single-Read flow cell on HiSeq 2500. For Round #3, #4, and #5, each round had 15 RNA samples and sequencing libraries of each round were pooled and sequenced on a Rapid Single-Read flow cell.

Figure S2. Example of a protocol description.

Directions for preparing Sample descriptions:

Note: At this time the website only accepts sample sets which use a three component mixture design (see https://doi.org/10.6028/NIST.SP.1200-23). Components and mixture proportions may be different from previous sample sets.

If your laboratory is entering a dataset that was derived from a sample set different from those listed (Step 3c), please use the following template to prepare a new sample set description (see **Figure S3** for example):

Sample Set Description

1. Laboratory/institution that prepared the sample set
2. Source of pure total RNA1:
    a. Tissue of origin
    b. Supplier
3. Source of pure total RNA2:
    a. Tissue of origin
    b. Supplier
4. Source of pure total RNA3:
    a. Tissue of origin
    b. Supplier
5. Fraction of each component in Mix1
    a. RNA1, RNA2, and RNA3
6. Fraction of each component in Mix2
    a. RNA1, RNA2, and RNA3
7. Aliquot contents
    a. Volume
    b. Concentration
8. Key to sample set (see **Figure S3** for example)

Send new or modified protocols to contact information located at:
https://edrn.nci.nih.gov/microrna.
A corresponding SampleID will be added to the analysis pipeline.

Prepared by (Institution)	NIST	
Component		
RNA1	Liver	Thermo Fisher Scientific, Ambion Human Total RNA
RNA2	Brain	Thermo Fisher Scientific, Ambion Human Total RNA
RNA3	Placenta	Thermo Fisher Scientific, Ambion Human Total RNA
Mix1 Composition		
RNA1 Fraction	0.25	
RNA2 Fraction	0.25	
RNA3 Fraction	0.5	
Mix2 Composition		
RNA1 Fraction	0.25	
RNA2 Fraction	0.5	
RNA3 Fraction	0.25	
Aliquot		
Volume (uL)	5	
Conc. (ug/uL)	1	
Sample	Tissue	Replicate
Sample_1	Mix1	Rep1
Sample_2	Placenta	Rep1
Sample_3	Liver	Rep1
Sample_4	Mix2	Rep1
Sample_5	Placenta	Rep2
Sample_6	Mix2	Rep2
Sample_7	Liver	Rep2
Sample_8	Placenta	Rep3
Sample_9	Brain	Rep1
Sample_10	Mix1	Rep2
Sample_11	Brain	Rep2
Sample_12	Brain	Rep3
Sample_13	Mix2	Rep3
Sample_14	Mix1	Rep3
Sample_15	Liver	Rep3

Figure S3. Tab-delimited text file with sample description and key.

Unique indentifier column(s)

Data column names match sample key

Mature_Name	Mature_ID	Sequence	Sample_1	Sample_2	Sample_3	Sample_4	Sample_5	•••	Sample_15
let-7a-5p	MIMAT0000062	UGAGGUAGUAGGUUGUAUAGUU	308794	218380	146529	458101	203879		229379
let-7b-5p	MIMAT0000063	UGAGGUAGUAGGUUGUGUGGUU	38757	11147	12472	66202	10415		20508
let-7c-5p	MIMAT0000064	UGAGGUAGUAGGUUGUAUGGUU	15903	4983	9180	27483	5161		14650
let-7d-5p	MIMAT0000065	AGAGGUAGUAGGUUGCAUAGUU	8789	2773	4052	14097	2498		6331
let-7e-5p	MIMAT0000066	UGAGGUAGGAGGUUGUAUAGUU	13146	13034	1876	16716	12177		2628
let-7f-5p	MIMAT0000067	UGAGGUAGUAGAUUGUAUAGUU	200431	98094	175731	312584	93595		244964
miR-15a-5p	MIMAT0000068	UAGCAGCACAUAAUGGUUUGUG	678	987	270	564	979		371
miR-16-5p	MIMAT0000069	UAGCAGCACGUAAAUAUUGGCG	19063	28897	6392	15462	33916		7691
miR-17-5p	MIMAT0000070	CAAAGUGCUUACAGUGCAGGUAG	5910	8158	5553	4226	10027		6334
miR-17-3p	MIMAT0000071	ACUGCAGUGAAGGCACUUGUAG	166	236	263	159	271		276
miR-18a-5p	MIMAT0000072	UAAGGUGCAUCUAGUGCAGAUAG	96	196	50	79	214		51
miR-19a-3p	MIMAT0000073	UGUGCAAAUCUAUGCAAAACUGA	664	757	802	590	790		1139
miR-19b-3p	MIMAT0000074	UGUGCAAAUCCAUGCAAAACUGA	10369	16077	8444	7520	19102		9784
miR-20a-5p	MIMAT0000075	UAAAGUGCUUAUAGUGCAGGUAG	9633	12363	11631	7568	14680		12420
miR-21-5p	MIMAT0000076	UAGCUUAUCAGACUGAUGUUGA	494834	761974	659397	407220	992713		529836
miR-22-3p	MIMAT0000077	AAGCUGCCAGUUGAAGAACUGU	94294	97890	119104	92275	93042		156600
miR-23a-3p	MIMAT0000078	AUCACAUUGCCAGGGAUUUCC	33740	54803	5921	20889	62051		7177
miR-24-1-5p	MIMAT0000079	UGCCUACUGAGCUGAUAUCAGU	412	[illegible]					[illegible]
miR-24-3p	MIMAT0000080	UGGCUCAGUUCAGCAGGAACAG							
miR-25-3p	MIMAT0000081	CAUUGCACUUG[illegible]							
[illegible]	[illegible]								

Figure S4. Tab-delimited text file with unique identifier column(s) for intersection with miRBase (ftp://mirbase.org/pub/mirbase/CURRENT/mature.fa.gz) and column names that match those in the sample key of the corresponding sample description file (see **Figure S3** for example).
